# Cognition In Later Life And Life Course Socioeconomic Determinants: Longitudinal Evidence From China (2011-2020)

**DOI:** 10.1093/geroni/igaf122.659

**Published:** 2025-12-31

**Authors:** Mengling Cheng

**Affiliations:** East China University of Science and Technology, Shanghai, Shanghai, China (People’s Republic)

## Abstract

Evidence showed that differences in later-life cognition were associated with variations in socioeconomic status (SES) in various life course periods. However, less is understood about the effects of SES over the life course and the underlying patterns. Guided by the life course perspective, this study examined the association between SES across childhood, adulthood, and older age and later-life cognition in China, testing four life course models: critical period, sensitive period, pathway, and accumulation. We used data from the China Health and Retirement Longitudinal Study (2011-2020, 17,000 participants). We measured cognition globally and across domains. We measured SES in childhood, adulthood, and older age, alongside a cumulative SES index. We performed multilevel growth curve models to test the four life course models. Results revealed that SES throughout the life course was associated with later-life cognition, with consistent evidence in global cognition and across cognitive domains. Findings supported the sensitive period, pathway, and accumulation models, but not the critical period model. In particular, older women and rural residents were more vulnerable to the adverse effects of socioeconomic disadvantages on cognition over time. Our study highlights the importance of addressing SES disparities across the life course to mitigate its impact on cognitive aging.

